# Absence of bladder cancer cells in surgical smoke from robot-assisted radical cystectomy: a prospective study

**DOI:** 10.3389/fruro.2026.1714844

**Published:** 2026-03-18

**Authors:** Kosuke Shibamori, Kohei Hashimoto, Ko Okabe, Takeshi Maehana, Tetsuya Shindo, Yuki Kyoda, Ko Kobayashi, Toshiaki Tanaka, Satoshi Takahashi, Naoya Masumori

**Affiliations:** 1 Department of Urology, Sapporo Medical University School of Medicine, Sapporo, Japan; 2 Department of Infection Control and Laboratory Medicine, Sapporo Medical University School of Medicine, Sapporo, Japan

**Keywords:** bladder cancer cell, dissemination, port site recurrence, RARC, surgical smoke

## Abstract

After robot-assisted radical cystectomy (RARC) for bladder cancer, urologists occasionally encounter distinct recurrences, including port site recurrence and peritoneal dissemination. We hypothesize that the surgical smoke generated during RARC could contain bladder cancer cells, potentially leading to dissemination. Initially, we examined the cytology of the exhaust smoke filters used during laparoscopic radical cystectomy; however, no cancer cells were detected. Subsequently, we conducted digital PCR analysis for the *PIK3CA* (E545K) gene mutation in surgical smoke collected through a water trap system during RARC. However, we were unable to detect any mutated genes. We subjected T24 bladder cancer cell line pellets to electrocoagulation vaporization and subsequently captured the surgical smoke through a vacuum system. However, we could not detect the *TERT* (C228T) mutation in the smoke. Consequently, we proceeded with exosome analysis of the smoke obtained from electrocoagulated pellets and the supernatant of T24 cells as the control. The exosome levels in smoke were significantly lower than that in controls. Based on these findings, we concluded that the surgical smoke produced during RARC does not contain cancer cells, genes, or exosomes.

## 1 Introduction

Robot-assisted radical cystectomy (RARC) and laparoscopic radical cystectomy (LRC) are standard treatments for muscle-invasive bladder cancer (MIBC) (1). However, distinctive recurrence patterns, such as port site recurrence or peritoneal dissemination, sometimes occur after RARC and LRC (2). Surgical smoke, defined as the gas generated by intraoperative manipulation, contains viral DNA (3). We hypothesize that bladder cancer cells could be spread by surgical smoke during RARC or LRC. Therefore, we conducted a prospective study to investigate this hypothesis.

## 2 Materials and methods

### 2.1 Patient selection

We examined the cytology of exhaust smoke filters as a routine procedure during LRC between October 2017 and March 2019. However, none of the patients showed positive cytological results for surgical smoke. Subsequently, as part of a prospective study, we attempted to capture cancer-associated genes carried by surgical smoke using the water trap method. We enrolled 28 patients who underwent RARC between October 2020 and March 2022. Ethical approval was granted by our institutional review board (no. 322-211), and informed consent was obtained from all participants. The primary outcome of this study was the detection rate of bladder cancer-associated genes.

### 2.2 Surgical smoke sampling and analysis of cancer-associated genes during RARC

**Figure 1:**
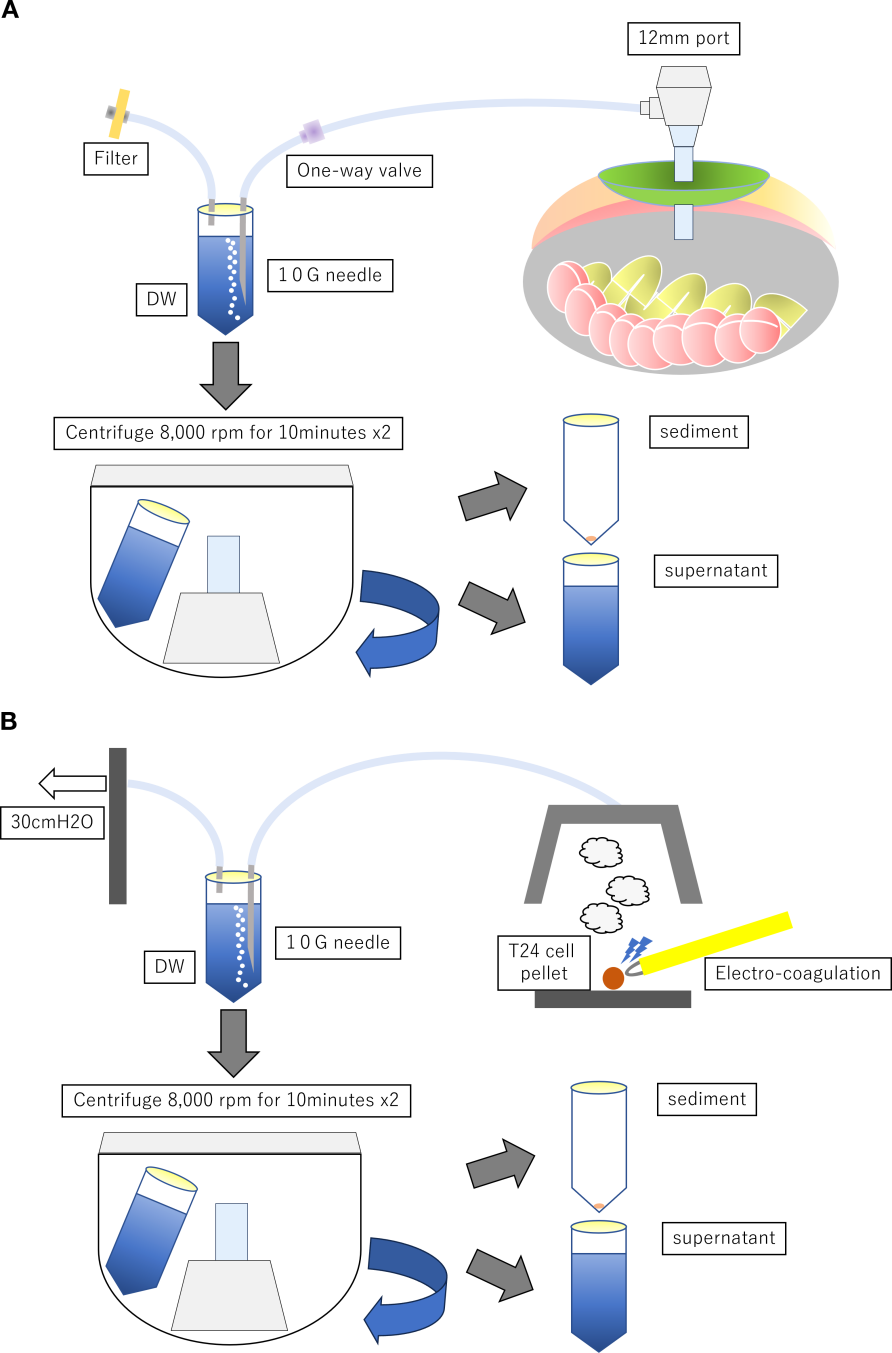
**(A)** Surgical smoke collection system during RARC. **(B)** Surgical smoke collection system during carbonization of T24 cell pellet.

During RARC, an extension tube was attached to the port. The tip was attached to a 10-G needle, which was inserted into a sterile centrifuge tube containing distilled water (DW). Another needle was bisected and inserted into a tube, with the tip exposed to the atmosphere through a sterile syringe filter (0.22 µm, PVDF, 33 mm; Millex-GV, Merck, Burlington, MA, USA) (**Figure 1A**). As the intraperitoneal cavity was highly pressurized by carbon dioxide gas, surgical smoke was eluted into DW like a water-sealing system. The bladder was placed inside an Endo Catch II and immediately extracted. After cystectomy and lymph node dissection, the tube was collected. DW was centrifuged twice at 8,000 rpm for 10 min each to separate the cellular components from the supernatant. Genomic DNA (gDNA) was purified from the cellular components using a QIAmp DNA Micro Kit (56304; Qiagen, Hilden, Germany). Cell-free DNA (cfDNA) was purified from the supernatant using a PAXgene cfDNA collection tube and a MagMAX Cell-Free Isolation Kit (A29319; Thermo Fisher Scientific, Waltham, MA, USA) according to the manufacturer’s protocol. Tumor and normal bladder tissues were collected immediately after the removal of the bladder from the body, and gDNA was extracted using a QIAmp DNA Mini Kit (51304; Qiagen, Hilden, Germany). Because we hypothesized that tumor DNA is at an extremely low level in surgical smokes, we could use the sample from each patient for one mutation detection kit in digital PCR. Thus, we focused on one specific tumor mutation. *TP53* was the most common tumor mutation of MIBC; however, there were multiple variants of mutation. We focused on *PIK3CA* E545K as the *PIK3CA* gene mutation for urothelial carcinoma is common and the mutation point is limited compared with *TP53* (4). The mutations detected using the *PIK3CA* E545K Digital PCR Mutation Detection Kit (Hs000000086_rm; Thermo Fisher Scientific, Waltham, MA, USA) were analyzed and identified using the QuantStudio 3D Digital PCR System (Thermo Fisher Scientific, Waltham, MA, USA).

### 2.3 Surgical smoke collection and analysis of the generated bladder cancer cell line pellets

The T24 bladder cancer cell line, which carries mutations in *TERT*, was passaged. T24 cells were cultured for 48 h and changed to serum-free medium at 80% confluence, followed by the collection of the supernatant and pellets. The pellets were completely carbonized using a Bovie cautery scalpel (*n* = 3; 21601010; Bovie, Clearwater, FL, USA). The upper air was aspirated at 30 cm H_2_O and trapped in a water seal of DW (5) (**Figure 1B**). The gDNA was purified from the supernatant and sediment, followed by the identification of *TERT* C228T mutations (Hs000000092_rm; Thermo Fisher Scientific, Waltham, MA, USA) using the method described in *Section 2.2*.

### 2.4 Exosome analysis

The exosome concentration in the surgical smoke generated from T24 cell pellet cauterization (*n* = 2) was quantified using a NanoSight LM10 instrument (FUJIFILM Wako Biosolutions Corporation, Richmond, VA, USA) using the T24 cell culture supernatant collected as described in *Section 2.3*. was used as the control (*n* = 1). The sample suspension was diluted sevenfold with Milli-Q water to make a 500-µl exosome dilution. Brownian motion observation images were captured five times for 60 s each, with the camera level set at 13. The particle size and concentration were calculated from image analysis. The threshold level of exosomes was set at 1.0 × 10 (8) particles/ml.

## 3 Results

Of the 28 patients with RARC, 22 (79%) were men and 13 (46%) received neoadjuvant chemotherapy. The median patient age was 73 years. There were 11, 7, 10, and 0 with pathological T ≤1, 2, 3, and 4, respectively. The results of the digital PCR analysis are shown in **Table 1**. None of the samples in the supernatant or sediment components from the surgical smoke had mutations in the *PIK3CA* gene, and the wild type was rarely identified. Similar to the surgical smoke of RARC, the surgical smoke generated by the T24 pellet did not contain mutations in *TERT*. In the exosome analysis, the surgical smoke had a notably low exosome level (mean particle size = 147 ± 28.1 nm, 0.52 ± 0.25 × 10^8^ particles/ml) compared with the T24 cell supernatant (mean particle size = 157 ± 11.4 nm, 2.38 ± 0.42 × 10^8^ particles/ml). As the control group consisted of only a single sample (*n* = 1), making variance estimation impossible, no statistical analysis was conducted.

**Table 1:** The results of digital PCR.

Surgical smoke collection method	DNA Collection Method		Total copies/µL	Target copies/µL	Target (%)
RARC (PIK3CA)	Normal tissue gDNA		532.2 (75.5-1495.5)	1.0 (0-24.3)	0.4 (0.0-8.8)
	Tumor gDNA		384.1 (0.1-1201.7)	0.3 (0-5.5)	0.2 (0.0-3.0)
	Surgical smoke	Supernatant (cfDNA)	0.3 (0.0-0.8)	0.0 (0.0-0.0)	0.0 (0.0-0.0)
		Sediment (gDNA)	0.6 (0.0-2.2)	0.0 (0.0-0.0)	0.0 (0.0-0.0)
T24 pellet (TERT)	Surgical smoke	Supernatant (cfDNA)	0.09 (0.0-0.26)	0.0 (0.0-0.0)	0.0 (0.0-0.0)
		Sediment (gDNA)	2.87 (0.24-8.1)	0.0 (0.0-0.0)	0.0 (0.0-0.0)
	Pellet	gDNA (control)	195.9	151.8	77.5

Average (range). PCR, polymerase chain reaction. RARC, robot-assisted radical cystectomy. PIK3CA, phosphatidylinositol-4,5-bisphosphate 3-kinase catalytic subunit alpha. TERT, telomerase reverse transcriptase. gDNA, genome DNA. cfDNA, cell-free DNA.

## 4 Discussion

In this study, we did not definitively detect cancer-associated mutation genes and exosomes in the surgical smoke from either the RARC or the cauterized T24 pellet procedure. Therefore, distinct recurrences after RARC are not directly attributable to surgical smoke. A randomized controlled trial comparing open radical cystectomy and RARC showed no significant differences in the recurrence rates and cancer-specific survival (6). However, the observed pattern of the first recurrence suggests an increased risk of local or abdominal recurrence following RARC. The incidence of atypical recurrence, such as port site metastasis and peritoneal carcinomatosis, after LRC or RARC ranges from 1% to 11% (7). Wei et al. demonstrated that higher levels of residual cancer cells in intraoperative pelvic washing were associated with recurrence in patients who underwent RARC (8). Several *in vivo* studies have implied that pneumoperitoneum itself may contribute to the development of peritoneal dissemination (9, 10).

Spillage of urine or tumor cells contained in metastatic lymph nodes may contaminate surgical instruments, a risk potentially exacerbated by pneumoperitoneum. Therefore, while avoiding cancer spillage and adhering to safety protocols, caution is required when handling these materials. Physicians should be aware that the risk of dissemination due to pneumoperitoneum is not completely eliminated during RARC.

This study has some limitations, including the examination of only a single mutation, the potential for inherent issues within the DNA collection methodology, and the lack of follow-up. Since statistical processing was not possible, we add that these results are preliminary and should be interpreted with caution. Due to the limited volume of the sample, we could not perform broader genomic profiling such as panel sequencing. Only a single gene could be analyzed in this study. Agarwal reported that frequent alterations in the metastatic lower urinary tract urothelial carcinoma cohort occurred in *TP53* (48%), *ARID1A* (17%), and *PIK3CA* (14%) (4). *PIK3CA* (E545K) was the third most frequent gene alteration, and the E545K mutation is overwhelmingly more common than in other sites. *TP53* was the most common gene alteration; however, it had multiple hotspots, making the detection of mutations inefficient. Therefore, we chose *PIK3CA* (E545K) mutation kits. We did not conduct a pre-screening of primary tumors to confirm whether they harbor the *PIK3CA* (E545K) mutation. This should be considered a significant limitation. Furthermore, there is no established or definitive method for capturing aerosolized DNA, as such techniques are not standardized. Moreover, the technique using T24 pellets does not fully correspond to the conditions encountered during surgery. Since amputation and ultrasonic-driven devices cannot be replicated, only a simple coagulation is reproduced. Yokoe et al. (5) successfully detected viral DNA in pneumoperitoneum using a water trap system. In the present study, we employed a similar approach; however, the system consistently yielded negative results, which supports the conclusion that tumor-derived DNA is absent in the pneumoperitoneum.

On the other hand, the negative results of this study suggest that this method is less sensitive than recent reports on urinary biomarkers indicate (8, 11). Urine-based liquid biopsies have dramatically improved the accuracy of molecular biological and cytological detection for diagnosing urothelial carcinoma and monitoring recurrence. Recent findings have demonstrated the efficacy of biomarker detection via liquid media, reporting high diagnostic accuracy for methods such as targeted deep sequencing of urinary DNA (8, 11). The sensitivity of detection systems using surgical smoke may be significantly limited due to dilution effects and collection efficiency. In addition, the heat generated during electrocautery may damage DNA, hindering the detection of target genes. Nevertheless, the methodology of this study should be highlighted for targeting “aerosolized genetic material” in a pneumoperitoneum environment. There are no studies demonstrating the presence of cancer tumor DNA in surgical smoke. We believe it is necessary to develop technologies that can reliably detect low concentrations of tumor-derived DNA. This can be accomplished by standardizing and improving surgical smoke collection methods while comparing their sensitivity to that of the existing liquid biopsy techniques using urine. We are currently investigating the measurement of circulating tumor DNA (ctDNA) in the blood before and during RARC. These consistent results could facilitate the future detection of cancer cells and cancer-derived genes in pneumoperitoneum. Future investigations are recommended to address this issue.

## 5 Conclusion

Cancer-associated mutated genes and exosomes were not detected in the surgical smoke produced during RARC and T24 pellet carbonization.

## Data Availability

All relevant data is contained within the article: The original contributions presented in the study are included in the article/supplementary material, further inquiries can be directed to the corresponding author/s.
